# Bias in Reporting of Genetic Association Studies

**DOI:** 10.1371/journal.pmed.0020419

**Published:** 2005-11-22

One of the tools in the scientist's armory for resolving a medical issue or consolidating a body of clinical trials is the systematic review of the published medical literature. This technique involves doing a literature search and critical appraisal of individual studies, and in addition, may also use statistical techniques to combine the results of these studies. One of the aims of such reviews is to assess and then, ideally, include all appropriate studies that address the question of the review. But finding all studies is not always possible, and researchers have no way of knowing what they have missed. But does it matter if some studies are left out?

It would definitely matter if the missing studies differed significantly from the included ones. And the worst-case scenario is that the accumulation of evidence might point to the wrong answer if the studies included are unrepresentative of all those that have been done.

Studies of publication bias have noted that papers with significant positive results are easier to find than those with nonsignificant or negative results. As a result, overrepresentation of positive studies in systematic reviews might mean that such reviews are biased toward a positive result. Publication bias is just one in a group of related biases, all of which potentially lead to overrepresentation of significant or positive studies in systematic reviews. Other types of bias include time lag bias (positive studies are more likely to be published rapidly); multiple publication bias (positive studies are more likely to be published more than once); citation bias (positive studies are more likely to be cited by others); and language bias (positive studies are more likely to be published in English).

In *PLoS Medicine*, John Ioannidis and colleagues have taken a closer look at bias in Chinese genetics studies. Research done in non-English-speaking countries has two outlets. A study might be published in English-language journals, which are usually indexed in major international bibliographic databases such as PubMed, or in domestic journals, many of which are not indexed in international databases. The Chinese literature is a prominent example of where domestic scientific journals are not catalogued in international databases. There is some evidence that the decision to publish in international versus domestic journals might be influenced by the results. For example, significant results are often published in international journals, whereas nonsignificant results appear in the local literature, resulting in a language bias—although, the reverse situation has also been described. [Fig pmed-0020419-g001]

**Figure pmed-0020419-g001:**
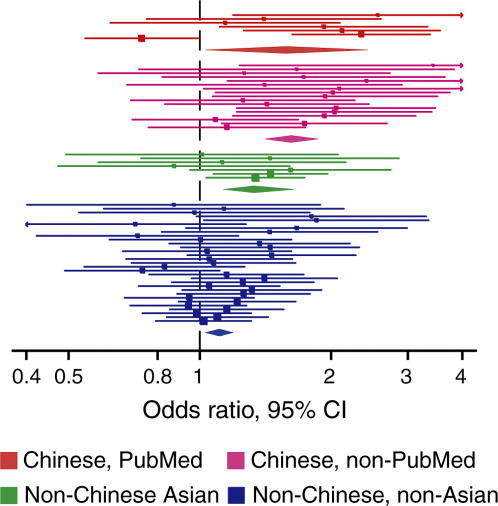
Different results in local literature studies

Genetics studies pose particular problems for impartial reporting. There are millions of polymorphisms in the human genome, and an exponentially increasing number of studies are trying to associate genetic polymorphisms with risk of disease or treatment outcomes. Selective publication might invalidate the overall picture of genetic risk factors.

The authors examined 13 gene–disease associations. Studies were more likely to be published when the disease was considered common in China. They found 161 Chinese studies on 12 of these gene–disease associations, only 20 of which were indexed in PubMed. Chinese studies had significantly more prominent genetic effects than non-Chinese studies, and 48% were statistically significant per se, despite their smaller sample size. Moreover, the largest, most exaggerated genetic effects were often seen in PubMed-indexed Chinese studies. Chinese studies usually appeared several years after their equivalent was first postulated in the world literature.

The larger genetic effects in Chinese studies are unlikely to reflect genuine heterogeneity and are more likely to do with publication bias operating within the Chinese literature, say the authors. It is possible that there was reluctance to submit and publish negative or inconclusive results when a large body of English-language literature has shown the presence of genetic effects. However, such “forced” confirmation negates the importance of independent confirmation of research results. This problem is probably not limited to the Chinese literature. These phenomena haven't been noted in molecular medicine before, but could become a serious problem in such a fast-moving field. Moreover, the inclusion of poor-quality research and additional selectively reported data may contaminate the better literature rather than provide a more accurate, comprehensive picture.

The findings have two broad implications. First, language bias might be important to consider in meta-analyses of observational studies, where its effect might be larger than its effect on randomized evidence. Second, because human genome epidemiology is a global enterprise, a comprehensive global view is important to help decipher artifacts from true genetic effects. The Chinese literature in particular will be essential for the evaluation of evidence on genetic risk factors. China is making rapid scientific progress in this field and joining in international collaborative projects, such as the Human Genome Project. To develop a global perspective, one way forward might be for all investigators working on the genetics of a specific disease to register with a common network, making it easier to trace additional unpublished or nonindexed data.

